# Superwettable Biosensor for Disease Biomarker Detection

**DOI:** 10.3389/fbioe.2022.872984

**Published:** 2022-03-28

**Authors:** Yun Jun Yang, Zhong Feng Gao

**Affiliations:** ^1^ Advanced Research Institute for Multidisciplinary Science, Qilu University of Technology (Shandong Academy of Sciences), Jinan, China; ^2^ Advanced Materials Institute, Qilu University of Technology (Shandong Academy of Sciences), Jinan, China

**Keywords:** biosensing, superwettability, bioinspired material, interface, detecting technologies

## Abstract

Bioinspired superwettable materials have aroused wide interests in recent years for their promising application fields from service life to industry. As one kind of emerging application, the superwettable surfaces used to fabricate biosensors for the detection of disease biomarkers, especially tumor biomarkers, have been extensively studied. In this mini review, we briefly summarized the sensing strategy for disease biomarker detection based on superwettable biosensors, including fluorescence, electrochemistry, surface-enhanced Raman scattering, and visual assays. Finally, the challenges and direction for future development of superwettable biosensors are also discussed.

## Introduction

The detection of potential disease biomarkers in patient samples is an important factor for screening and early diagnosis of diseases, such as cancer (Karachaliou et al., 2015; Wu and Qu, 2015). The abnormal expression of genes, proteins, tumor-related mRNA, exosomes, and circulating tumor cells is closely associated with the occurrence of tumor and has been generally considered specific biomarkers to evaluate the development stage of cancer (Hanahan and Weinberg, 2000; Meng et al., 2021). Recently, tremendous efforts in the field of the disease biomarker biosensing method provide rich diagnostic and prognostic information for disease management (Seferos et al., 2007; Gong et al., 2021; Liu et al., 2022). Among them, the use of superwettable property-based biosensors is an emerging research field. Superwettable surfaces, such as lotus leaf-inspired superhydrophobic surface, Namib Desert beetles-inspired patterned superwettable surface, and *Nepenthes* pitcher plant-inspired slippery surface, are commonly used for the development of novel superwettable biosensors. These bioinspired surfaces exert unique liquid-repellent performance with large contact angle, decreasing the contact area between the droplet and surface (Dong et al., 2018; Sun et al., 2021). The remarkable wetting behavior brings several merits, such as remarkable evaporation-enrichment effect and new insights into visual biosensing. Superhydrophobic and slippery surfaces provide an effective and simple strategy to concentrate the analyte inside the droplet and improve spot homogeneity, promising for the fabrication of sensitive biosensor. The patterned superwettable surface has the feature to anchor the droplet, which holds the potential for the high-throughput biosensor.

It is reported that worldwide *in vitro* diagnostics market investments are growing every year, implying that biomedical diagnostic tools are playing key roles in disease diagnosis and human health assessment (Sassolas et al., 2008; Collins et al., 2021). The eventual aim of these endeavors is the development of point-of-care testing (POCT) devices with high selectivity, sensitivity, accuracy, and real-time detection for real sample analysis. Compared to the conventional methods using solution systems, the superwettable biosensing strategy used the droplet as the reaction system, which is promising for POCT applications due to their flexibility, easy-to-use, portability, and short sample processing time (Chen et al., 2020; He et al., 2021; Zhu et al., 2021). To realize this goal, researchers developed various versatile and robust superwettable biosensors that meet the requirement of clinical patient sample assays. In recent years, biosensing methods including fluorescence, electrochemical, surface-enhanced Raman scattering (SERS), colorimetry, and visual assays are widely employed in analytical chemistry. Integrations between these biosensing strategies and superwettable surfaces have been put forward by researchers in quest of biomarker detection.

**FIGURE 1:**
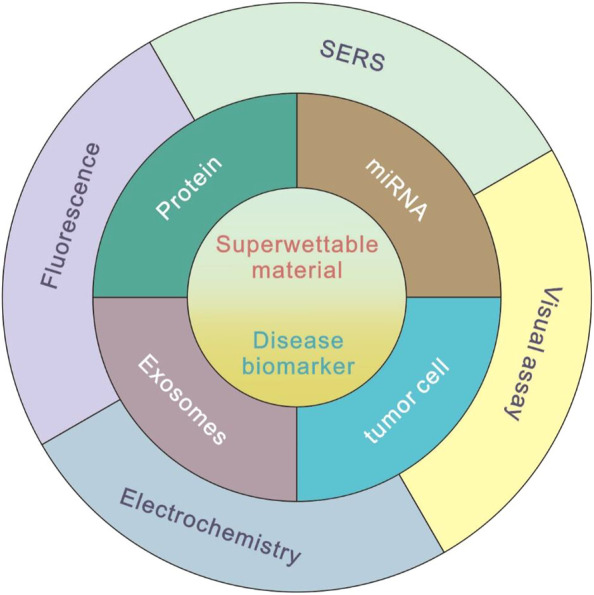
Commonly used methods of superwettable biosensors for disease biomarker detection.

In this mini review, we summarized the recent progress of biosensing applications based on bioinspired superwettable surfaces, such as superhydrophobic surfaces, patterned wettable surfaces, and slippery surfaces. Various detecting techniques, including fluorescence, electrochemical, SERS, colorimetric, and visual methods are combined, respectively, with different superwettable surfaces. The application in the field of biomarker detection is described in detail. By introducing the commonly used biosensing methods, such as fluorescence, electrochemistry, SERS, and visual assays, the superwettable biosensors have been demonstrated to be a useful platform in the field of disease biomarker detection (Figure 1). Finally, the highlights and challenges of superwettable biosensors for biomarker detection were discussed.

## Different Types of Superwettable Biosensors

### Fluorescence-Based Superwettable Biosensor

Fluorescent methods have attracted increasing attention because they do not require costly or sophisticated equipment and have been widely used in portable, *in situ*, and rapid detection (Hou et al., 2015). However, it has been reported that the detecting targets are dispersed in large volumes with the weak signal and low signal-to-noise ratio which cannot be detected effectively (Yan et al., 2014; Zhan et al., 2015). To solve this problem, droplet evaporation enrichment was developed to concentrate the targets from highly diluted solution to an area-confined domain to increase the effective contact frequency between the signal probes and targets (Gao et al., 2009).

Zhang et al. pioneered such an approach that the superhydrophobic TiO_2_ surface was designed with spotting superhydrophilic microwells (Xu et al., 2015). By the silane chemistry process, the capture probe was attached onto the superhydrophilic microwell. As the miRNA-141, a biomarker of prostate cancer, and FAM-labeled probe were introduced subsequently, the probes could be enriched and specifically recognized by the immobilized capture probe, resulting in the formation of the sandwich structure and exponential enhanced fluorescence intensity. This superwettable biosensor was realized for sensitive and selective detection of miRNA-141 with a low limit of detection (LOD) of 88 pM (Xu et al., 2018). This strategy has been applied for ultrasensitive detection of different cancer biomarkers, such as free prostate-specific antigen (PSA) (Chen et al., 2018) and mRNA (Hu et al., 2017). For a comprehensive understanding of the development and biosensing application of superwettable micropatterns, several high-quality reviews can be found in the literature (Xu et al., 2019; Wang et al., 2021).

**FIGURE 2:**
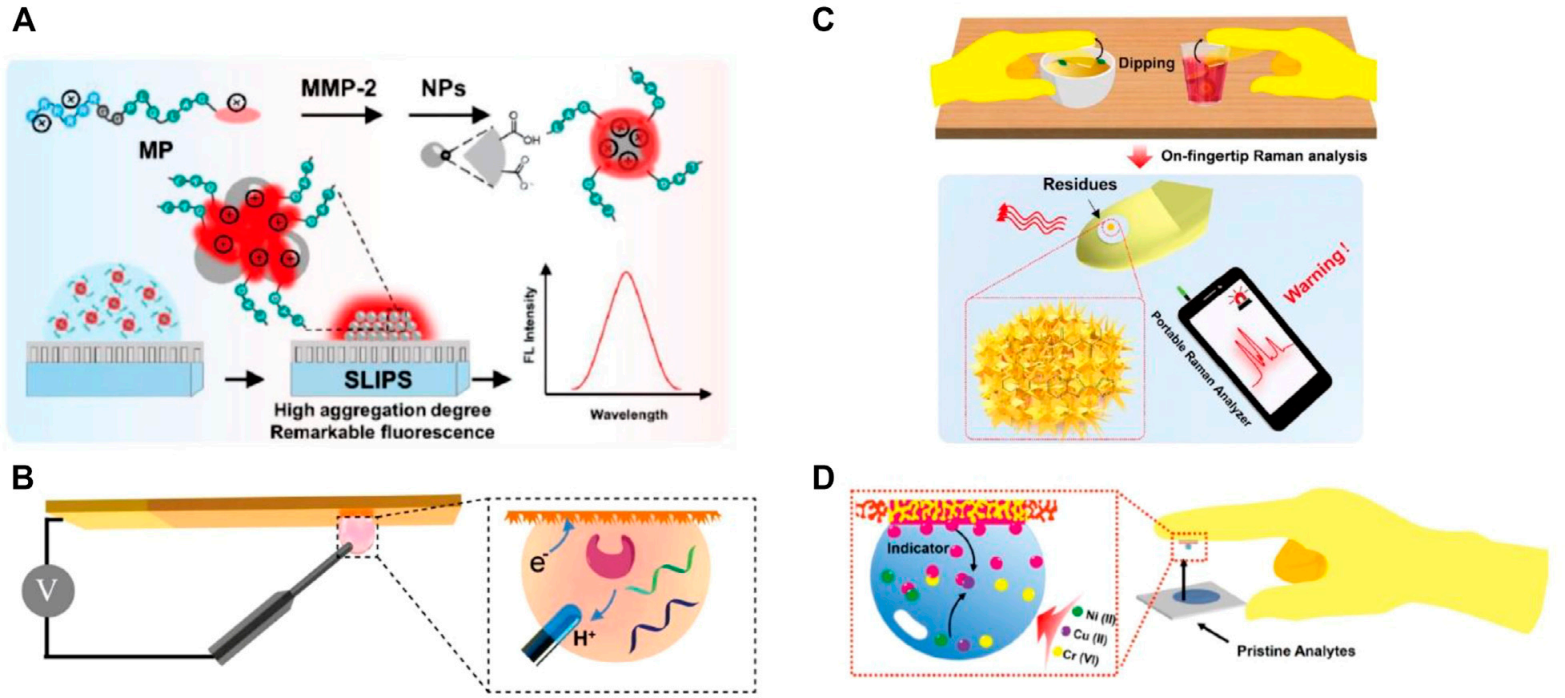
**(A)** Sensing process of peptide-conjugated AIEgen for quantitative detection of MMP-2 secreted from cells on the slippery surface (Wu et al., 2021). **(B)** Superwettable electrochemical microchip toward PSA detection (Xu et al., 2018). **(C)** Tape-based SERS superwettable sensors for the detection of food contaminants in an on-hand way (He et al., 2020b). **(D)** Design of the superwettable tapes for the colorimetric monitoring of heavy metals (He et al., 2018).

With the signal probe condensed after droplet evaporation, the aggregation-induced quenching effect might present, leading to the inaccurate analysis and even false-positive results. To address this problem, Lou et al. proposed an aggregation-induced emission (AIE) luminogen-based fluorescent method for the detection of matrix metalloproteinase-2 (MMP-2) tumor marker on slippery lubricant-infused porous substrates (SLIPSs) (Figure 2A). This SLIPS method obtained a low LOD of 3.7 ng/ml, which has been successfully used for detecting the MMP-2 secreted by tumor cells directly (Wu et al., 2021).

Based on the aforementioned discussion, fluorescence-based superwettable biosensors offer sensitive and accurate features to concentrate the sample and amplify the fluorescent signal, making them promising for the sensitive biomarker detection. However, the current methods mainly rely on the fluorescent microscope for laboratory measurements, which is cumbersome and not suitable for POCT application. The future direction should focus on the development of a portable fluorescent method, especially the smartphone-based superwettable biosensing method.

### Electrochemistry-Based Superwettable Biosensor

As an ultrasensitive and universal analytical method, electrochemical assays have significant advantages including low cost, rapid response, simple operation, and high sensitivity, and they have been widely considered the powerful tool for biosensing (Hasanzadeh et al., 2017; Mani et al., 2021). Combining the characteristics of the superwettable surface with the merits of the electrochemical system, prominent performances have been realized in the following examples.

Xu et al. reported a nanodendritic electrochemical biosensor based on superhydrophilic microwells on a superwettable microchip for the selective and sensitive determination of prostate cancer biomarkers such as miRNA-141 (LODs = 0.8 nM), miRNA-375 (LODs = 0.8 nM), and PSA (LOD = 1.0 pM) (Xu et al., 2018) (Figure 2B). Li’s group developed a refreshable electrochemical biosensor with an excellent self-cleaning property by casting superhydrophobic conductive polydimethylsiloxane (PDMS) and multiwalled carbon nanotubes nanocomposite onto a glassy carbon electrode (Zhu et al., 2017). By recording HRP-dependent electrochemical signals, a tumor marker, namely, carcinoembryonic antigen has been successfully detected by this method with a wide dynamic range from 0.1 to 100 mg/ml, and the detection limit is as low as 0.041 ng/ml. To meet the requirement for personal healthcare management at home, Zhang’s group developed several portable electrochemical micro-workstation platforms for detecting biomarkers of disease, such as glucose (Song et al., 2020b), miRNA (Song et al., 2019), and multiple Alzheimer’s disease biomarkers (Song et al., 2020c; Liu et al., 2022). These smart electrochemical biosensors demonstrated significant performance on cloud data management and multichannel detection, indicating great potential for remote detection and portable high-throughput biomedical applications in future.

These current electrochemical methods based on the superwettable surface demonstrate a great perspective in biosensing. The current superwettable biosensor cannot be reused. To overcome this problem, nucleic acid probes with regenerated conformation can be considered to construct sensitive superwettable biosensors.

### SERS-Based Superwettable Biosensor

Due to the significant advantages including small testing volume, rapid output, and high sensitivity, the SERS has been widely applied in various research fields such as sensing, bioimaging, food analysis, and environmental monitoring (Cardinal et al., 2017; Lin and He, 2019; Gao et al., 2021; Lin et al., 2021). To achieve significant performance, the SERS substrate incorporated with superwettability become an ideal candidate to access not only abundant hot spots for acquiring excellent sensitivity but also equally distributed hot spots for generating a stable signal.

Di Fabrizio et al. reported an interesting example for the direct detection of exosomes by SERS with a superhydrophobic array of silicon micropillars decorated with silver nanostructures (Tirinato et al., 2012). They found that exosomes from tumor colon cells show a high presence of RNA, whereas exosomes obtained from healthy colon cells display a high presence of lipid signals. Suarasan and coworkers also reported a superhydrophobic plasmonic biosensor for SERS-sensitive detection of exosomes with only 0.5 μL testing sample. PDMS was used to fabricate the superhydrophobic substrate with nanobowl and microbowl structures by the soft lithography method. Then, silver nanoparticles were grown *in situ* to impart SERS-enhancing properties (Suarasan et al., 2020). Yang et al. synthesized Fe_3_O_4_/Au/Ag nanocomposites and proposed a magnetically assisted SERS method to detect adenosine traces in clinical urine samples from lung cancer patients (Yang et al., 2014). This label-free method showed excellent sensitivity down to 1 × 10^–10^ M. Feng et al. developed an automatic deep learning-based superhydrophobic SERS platform for label-free detection of 695 clinical serum samples including 321 breast cancer patients, 77 leukemia M5 patients, 94 hepatitis B virus patients, and 203 healthy volunteers. This method demonstrated a high diagnostic accuracy (98.6%), which is promising for rapid, high-throughput, and label-free screening for cancer (Lin et al., 2021). With various designs of the superhydrophobic substrate, SERS-based superwettable biosensors have also been used to detect diverse cancer biomarkers, such as miRNA (Song et al., 2018; Song X. et al., 2020), extracellular vesicles (Suarasan et al., 2020), and peptides (Perozziello et al., 2014). These methods provide enormous potential to construct POCT devices for the early diagnosis of cancer. In addition, Zhang et al. proposed Au nanodendrites-functionalized superwettable microwells on the conductive carbon tape surface (He et al., 2020b). This sensor realized early-warning SERS detection of various food contaminants, such as thiabendazole, thiram, and Sudan-1, from real samples (Figure 2C).

These investigations provided a sensitive and accurate solution for coupling superwettable surface with SERS biosensing. However, the aggregation of targets is accompanied by the aggregation of contaminants during the droplet evaporation process, which is not desired in biosensing. To address this issue, pretreatment of samples is necessary before detection.

### Colorimetric/Visual Method-Based Superwettable Biosensor

There have been extensive endeavors dedicated to the development of a quantitative visual method in the context of cancer biomarker assays. Colorimetric assay is a classic visual strategy for detection due to its equipment-free, simple, and rapid advantages (Sabela et al., 2017; Xu et al., 2017). As superwettable behaviors, such as contact angle and rolling/sliding angle performance, are the most obvious and direct characteristics of the superwettability, they have been emerged as a novel visual strategy for biosensing.

Superwettability is typically used to develop paper-based analytical devices (PADs) with superhydrophilic microwells on a hydrophobic wax substrate. Whitesides et al. pioneered the first PADs, leading the trend of PADs for diverse applications (Martinez et al., 2007). For example, Chen et al. reported a highly sensitive colorimetric method for prostate-specific antigen (PSA) diagnosis using gold nanoparticles labeled with biotinylated poly (adenine) ssDNA sequences and streptavidin–horseradish peroxidase for enzymatic signal enhancement (Huang et al., 2018). They realized a detection limit down to 10 pg/ml for PSA detection within 15 min of experimental operation. Hou et al. reported a disposable colorimetric assay based on droplet array that has been constructed from diverse chemo-responsive colorants. This rapid, small, inexpensive, non-invasive, and visualized droplet array achieved an accuracy of at least 90% and can be used as a powerful tool for early screening of lung cancer (Zhong et al., 2018). Using flexible tapes, Zhang’s group established a superwettable colorimetric biosensor for on-site heavy metals monitoring (He et al., 2018). They achieved quantitative colorimetric detection of multiplex heavy metal ions including copper, chromium, and nickel by the naked eye (Figure 2D). Furthermore, they applied a smartphone to acquire colorimetric signals for semiquantitative detection of routine urine biomarkers (glucose, nitrite, protein, and phenylpyruvate) (He et al., 2020a) and sweat biomarkers (pH, chloride, glucose, and calcium) (He et al., 2019). The tape-based superwettable biosensors show significant merits including user-friendly, POCT potential, and favorable screening for the early disease warning toward the clinical patients. We presented a contact angle-based visual biosensing method based on the pH-responsive superhydrophobic surface. PSA can be detected with a low LOD of 3.2 pg/ml by analyzing the contact angle (Gao et al., 2019). The contact angle-based method is suitable for color-blind and color-weak individuals. Another method suitable for color-blind and color-weak individuals is sliding angle-based visual detection, in which by tuning the hydrophobic interaction between DNA and organogel, miRNA 21 can be detected by analyzing the sliding angle (Gao et al., 2020). As the superwettable performance of these biosensors was hardly influenced by temperature, elevation, and even droplet color, it has significant potential to numerous users, especially to those color-blind/weak people.

These current wetting behavior-based visual assays have direct implications for developing simple, rapid, and low-cost strategies for biomarker detection. However, the small changes of contact angle and rolling/sliding angle cannot be discriminated by the naked eye. Thus, it is desirable to develop a smartphone-based digital method for the visual detection.

## Conclusion

In summary, the recent progresses in superwettable biosensors for the detection of different biomarkers are briefly summarized, including the strategies of fluorescence, electrochemical, SERS, and visual assays. With continuing interdisciplinary technology and research progress, endless bioinspired nanomaterials and detection strategies will be introduced in the biosensing platforms. To note, several challenges also remain to be addressed in future developments. First, as almost applications presented in this review are mainly single target detection, developing a high-throughput superwettable biosensor with a multifunctional testing area would be more challenging and practical for future application. Second, the external stimulations including contamination and destruction may influence the wettability of the surface, leading to poor repeatability and credibility of such superwettable biosensors. Thus, long-surviving wettable surfaces are urgently required for practical application under extreme and complex biomedical conditions. Finally, the specificity of superwettable biosensing should be highlighted. Because the superwettable surfaces are preferred to interact with nonspecific targets by hydrophobic interaction and electrostatic interaction in complex environments such as cell matrix and blood, leading to the conformational change, recombination, and even oxidation of surface molecules. To avoid false-positive results, it is necessary to address the specificity for detection. We hope that this mini review will provide current insights and inspire researchers to investigate toward solving these existing problems and explore the superwettable biosensors as simple and commercialized devices for disease biomarker detection.
